# Sustainability of wild plant use in the Andean Community of South America

**DOI:** 10.1007/s13280-021-01529-7

**Published:** 2021-04-16

**Authors:** Laura Kor, Katherine Homewood, Terence P. Dawson, Mauricio Diazgranados

**Affiliations:** 1 Natural Capital and Plant Health Department, The Herbarium, Royal Botanic Gardens Kew, Richmond, TW9 3AE UK; 2 Department of Geography, Bush House NE, King’s College London, London, WC2B 4BG UK; 3 Anthropology Department, University College London, Gower Street, London, WC1E 6BT UK; 4 Natural Capital and Plant Health Department, Royal Botanic Gardens Kew, Wakehurst Place, Ardingly, RH17 6TN UK

**Keywords:** Conservation-through-use, Ethnobotany, Natural resource use, NTFP, Plant conservation, Useful plants

## Abstract

Overexploitation is the second biggest driver of global plant extinction. Meanwhile, useful plant species are vital to livelihoods across the world, with global conservation efforts increasingly applying the concept of ‘conservation-through-use.’ However, successfully balancing conservation and biodiversity use remains challenging. We reviewed literature on the sustainability of wild-collected plant use across the countries of Colombia, Ecuador, Peru, and Bolivia—a region of global importance for its biological and cultural richness. After applying defined search terms and a two-stage screening process, 68 articles were reviewed. The numbers which reported sustainable, unsustainable, or context-dependent outcomes were relatively even, but national differences emerged. Through narrative synthesis, we identified five key, reoccurring themes: plant biology; land tenure; knowledge, resource, and capacity; economics and market pressures; and institutional structures, policy, and legislation. Our results show the need for flexible, context-specific approaches and the importance of collaboration, with bottom-up management and conservation methods involving local communities and traditional ecological knowledge often proving most effective.

**Supplementary Information:**

The online version contains supplementary material available at 10.1007/s13280-021-01529-7.

## Introduction

Plants underpin all terrestrial ecosystems on earth. They provide the structure and resources needed for other organisms to survive and support a multitude of essential human uses and ecosystem services (Millennium Ecosystem Assessment 2005; Giam et al. 2010).

There are more than 40 000 reported useful plant species—“documented as fulfilling a particular need for humans, animals, or the wider environment” (Canteiro et al. 2016; Diazgranados et al. 2020). However, two in five plant species across the world are estimated to be at risk of extinction, prompting global conservation efforts (Brummitt et al. 2015; Corlett 2016; Nic Lughadha et al. 2020).

The Aichi Biodiversity Targets for 2011–2020 (CBD 2018) and the Global Strategy for Plant Conservation (GSPC) include targets regarding the conservation of crop wild relatives and other socio-economically valuable plant species (CBD 2012). Additionally, Target 12 of the GSPC concerns sustainable sourcing of wild-harvested plant products. Despite this, studies indicate that conservation targets for useful wild plant species have not been met (Khoury et al. 2019) and overexploitation continues to be a major driver of plant loss (Brummitt et al. 2015). The draft post-2020 global biodiversity framework places a greater emphasis on conserving biodiversity “for the benefit of planet and people” (CBD 2020b).

### Natural resource use and conservation

Traditional conservation efforts were often based on the separation of human societies and nature. This led to exclusionary protected areas, with many instances of restricted natural resource use and the eviction of local communities (Tuxill and Nabhan 1998; Brockington 2002; Robbins 2012). While the importance of biodiversity to humans has gained increasing recognition, so too has the importance of understanding the human dimensions of conservation and community involvement.

The concept that biodiversity conservation can be incentivized through the use of wild natural resources is often referred to as ‘incentive-driven conservation’ or ‘conservation-through-use’ (Freese 1997; Hutton and Leader-Williams 2003; Cooney 2007). This has numerous potential benefits, including the less destructive alternative that resource harvesting provides compared to other land uses; its contribution to the welfare of local communities; and its role in increasing the perceived value of habitats, incentivizing protection (Bennett 2002; Bussmann 2002). However, the conservation-through-use approach assumes sustainable resource use—“in a way and at a rate that does not lead to the long-term decline of biological diversity” (UN 1992)—an outcome often difficult to achieve in practice (Hutton and Leader-Williams 2003).

Despite the debates surrounding the concept, conservation-through-use is applied in conservation programs and research across the world. A *Policy Statement on Sustainable Use of Wild Living Resources* was published in 2000 by the International Union for Conservation of Nature (IUCN), placing it firmly in the conservation toolbox (IUCN 2000).

The increasing application of conservation-through-use approaches to plant and habitat conservation has resulted in a growing body of relevant literature (De Jong et al. 2000; Marshall et al. 2006). Some theoretical reviews and critiques have been published, particularly in the context of non-timber forest products (NTFPs) (Bennett 2002; Newton 2008). However, large-scale comparative reviews of studies relevant to the sustainability of wild plant use are lacking. While overexploitation of plants is the second biggest driver of extinction after habitat loss, plant use is also vital to the livelihoods and worldviews of many rural and indigenous populations globally (Newton 2008).

### The Andean community

The tropical Andean countries of Colombia, Bolivia, Peru, and Ecuador support a significant proportion of global biodiversity. Colombia, Peru, and Ecuador are recognized as ‘megadiverse’ (UN-WCMC 2014) and the region includes the biodiversity hotspots of the Chocó-Darien and Tropical Andes (Myers et al. 2000), the latter considered the global epicenter for biodiversity (Gonda 2020). These countries are party to the Andean Community (CAN) trade bloc and jointly formed the Andean Regional Biodiversity Strategy (Guinand and Gutiérrez 2005). Additionally, they are all signatories of the Convention on Biological Diversity’s (CBD) Nagoya Protocol.

The area’s high biological and cultural diversity has led to many ethnobotanical studies (Albuquerque et al. 2013; Paniagua-Zambrana and Bussmann 2020) and the formation of GELA (Grupo Etnobotánico Latinoamericano). Meanwhile, environmental pressures and conservation efforts have been widely documented, including conservation-through-use (Bussmann 2002; Cuoco and Cronan 2009; Fadiman 2019). However, there remains a lack of comparative studies which draw together and evaluate existing management and conservation of useful wild plant species in the area.

This study aimed to review literature on the sustainability of wild plant use across the Andean Community (Colombia, Peru, Ecuador, Bolivia). Our focus on wild-collected species reflects the conservation targets of the GSPC and refers to plants collected in natural or semi-natural ecosystems, as opposed to intensely cultivated plantations such as agricultural or silvicultural systems (Heywood 1999). We included studies that investigated the sustainability of existing use, management, and collection practices (Sheldon et al. 1997); projects which were implemented specifically for the conservation of useful plant species; and relevant comment articles and reviews.

The main objectives of this review were to summarize and evaluate (1) the characteristics of studies on in situ conservation and management of useful wild-collected plant species in the Andean Community; (2) factors identified as driving unsustainable harvest or loss of useful wild-collected plant species; and (3) outcomes and recommendations for sustainable management. Based on the CBD’s definition of *sustainable use* (1992), we define it in this paper as the “use of wild plants in a way and at a rate that does not lead to the long-term decline of botanical diversity, thereby maintaining traditional knowledge associated with its use and its potential to meet the needs and aspirations of present and future generations.” Based on results, key themes are highlighted and recommendations for conservation and management are proposed.

## Methods

### Literature searches

We used a systematic search strategy for this study (Pullin and Stewart 2006), forming search terms by combining three main concepts: location; useful plant species; and conservation or sustainable management. Relevant alternative expressions and wildcard operators were determined through search term scoping and merged with Boolean operators to form search strings in English and Spanish (Table S1). We performed bibliographic searches in the Scopus and Web of Knowledge databases in April to May 2020.

Results were imported to the reference management software Endnote (version X9) and duplicates deleted. We included both primary and secondary literature results.

### Selection of literature

**Fig. 1:**
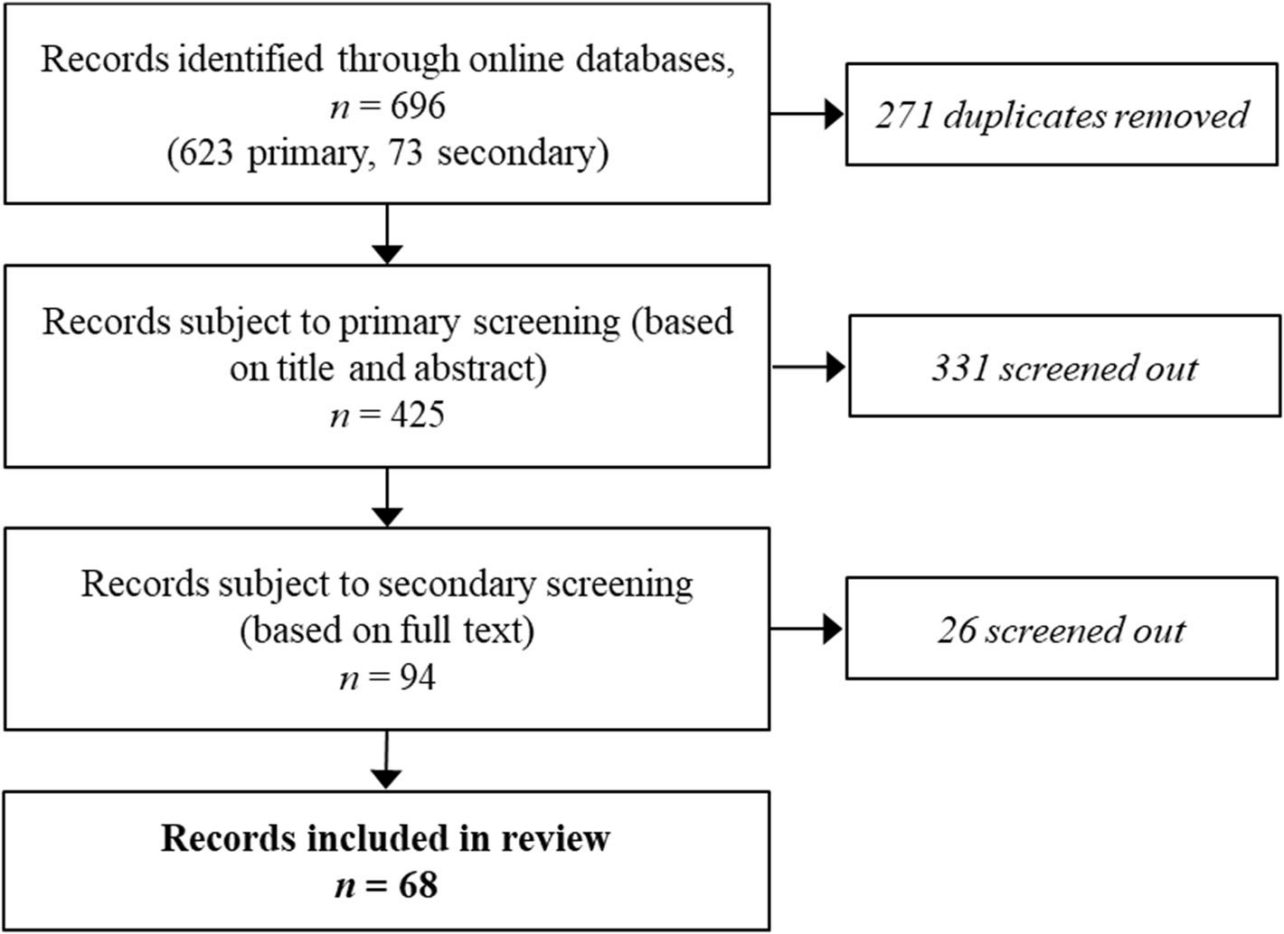
Flowchart of search and screening results for the conservation and management of useful wild plant species in the Andean Community

All results from the bibliographic search were subject to a two-stage screening process. Primary screening was based on titles and abstracts, with the full texts of resulting references screened in the second stage (Fig. 1). We applied set eligibility criteria, excluding studies which did not meet any one or more of the following:

1. Full text: the whole text of the reference could be sourced. This included searching online, in accessible libraries, and contacting authors where necessary.
2. Scientific merit: the document was subject to a form of peer-review to ensure validation of the academic work, including journal articles, book chapters, reports by governmental and non-governmental organizations (NGO), policy documents, botanic garden data, and PhD theses.
3. Location: the study was at least partially undertaken in Colombia, Ecuador, Peru, or Bolivia.
4. Ethnobotanical interest: the study included information on useful wild-collected plant species.
5. Biodiversity conservation relevance: the study was relevant to sustainable resource management or in situ conservation of wild species. This included assessments of the drivers of useful wild plant loss, existing management practices, introduced conservation measures, protected areas, policy and regulation, and conservation recommendations.

In defining ‘wild plants,’ we referred to the four phases of domestication for field and tree crops defined by Harris and Hillman (1989) and Wiersum (1997), respectively. We included studies on systems falling within definitions of domestication phases one and two, with phases three and four excluded (Wiersum 1997):

Phase 1. Harvesting of useful wild plants by gathering/collection in uncontrolled, open access, natural habitats (included in review).

Phase 2. Systematic collection of wild plants with tending of valued species in natural habitats, or selective cultivation of useful species by artificial in situ regeneration with small-scale land clearance (included in review).

Phase 3. Cultivation of selected species in artificially established plantations or systems with larger-scale land clearance and systematic tillage (not included in review).

Phase 4. Cultivation of domesticated plant species as crops or in intensively managed plantations (not included in review).

### Data extraction and synthesis

Due to the nature of the research questions and heterogeneity of the studies involved, we applied narrative synthesis in the review (Pullin and Stewart 2006). A standardized data extraction table was developed to record information for each study, including study countries, biomes, useful plant categories, focal taxa, social communities, and an assessment on whether existing sustainable harvesting or successful management intervention was achieved (Table S2).

Useful plants were categorized grouped within ten categories of use, in accordance with the *World Checklist of Useful Plant Species* (Diazgranados et al. 2020), applying a simplified version of the ‘Level 1 States’ described in the *Economic Botany Data Collection Standard* (EBDCS) (Cook 1995) (Table 1). Categories were allocated based on the uses focused on by each study’s authors, rather than all the known uses of the species. Where studies did not have a focal category or categories, but gave an overview of all uses, this field was described as ‘all.’ The EBDCS was developed as part of the International Working Group on Taxonomic Databases (TDWG) and adopted as a standard by the International Union of Biological Sciences (TDWG 1995). Despite the EBDCS being successfully applied by ethnobotanists investigating plant uses in many parts of the world (Grace et al. 2020; Tellez et al. 2020; Ulian et al. 2020), it is not universally accepted, and some modifications have been proposed (Ulian et al. 2017; Diazgranados et al. 2018). Other related standards exist, such as the IUCN’s *General Use and Trade Classification Scheme* (IUCN 2020), developed to record the end uses of wild-harvested species. It has several overlapping categories with the Level 1 States of the EBDCS, but it is also applied to wild animals and therefore less specific to plant use. Some authors continue not to use a standardized schema (Stepp and Thomas 2010), or prefer to apply standards developed in other fields to categorize more specific uses, such as the World Health Organization’s (WHO) International Classification of Diseases (ICD) in studies of plant medicinal uses (Heinrich et al. 2009; Staub et al. 2015). We chose to apply the modified version of the EBDCS following its use for the latest State of the World’s Plants and Fungi report 2020 (Antonelli et al. 2020).

**Table 1:** The categories used to classify useful plants investigated in each study reviewed, as defined in Diazgranados et al. (2020)

Level 1 category	Description
Animal food (AF)	Forage and fodder for vertebrate animals
Environmental uses (EU)	Examples include intercrops and nurse crops, ornamentals, barrier hedges, shade plants, windbreaks, soil improvers, etc.
Fuels (FU)	Wood, charcoal, petroleum substitutes, etc. separated from materials because of their importance
Gene sources (GS)	Wild relatives of major crops which may be valuable for breeding programs
Human food (HF)	Food and beverages for humans only
Invertebrate food (IF)	Plants eaten by invertebrates which are useful to humans (e.g., silkworms)
Materials (MA)	Woods, fibers, cork, cane, tannins, latex, gums, etc. and their derived products
Medicines (ME)	Both human and veterinary
Poisons (PO)	Plants which are poisonous to vertebrates and invertebrates, both accidentally and usefully (e.g., for hunting and fishing)
Social uses (SU)	Plants used for social purposes not definable as food or medicines. Such as smoking materials, hallucinogens and psychoactive drugs, contraceptives and abortifacients, and plants with ritual or religious significance

Biomes and ecoregions were categorized by comparing the study location or locations against the *Terrestrial ecoregions of the world: a new map of life on Earth* (Olson et al. 2001). Combinations were possible for several of the fields in the data extraction table.

Based on the results and conclusions of the authors, we classified whether the harvesting regime or conservation intervention investigated was sustainable. Where sustainable harvesting was found to occur under only certain contexts, this was classified as ‘variable’ (Table S2).

We characterized studies against key variables such as country, year of publication, and plant use. We conducted Chi-square tests and Fisher’s exact tests in R (R Core Team 2020) to assess statistical variations in publication trends and results. Qualitative analyses were then undertaken, based on drawing out key information, results, and recommendations relevant to three research questions:

1. What are the key drivers of sustainable and unsustainable harvesting and maintenance or loss of useful wild plant species?
2. What existing management practices or conservation interventions have been assessed and how successful are they in sustainable use?
3. What recommendations have been made to improve conservation and management outcomes?

## Results

The preliminary search returned 425 unique records across the two databases. Following the first stage of screening, we reviewed the full text of 94 records against the eligibility criteria, with 68 records included in the review (Fig. 1; Table 2).

**Table 2:** Characteristics of studies included in the review on the conservation and management of useful wild plant species in the Andean Community

Reference	Year published	Reference type	Study countries^a^	Biomes^b^	Useful plant categories^c^	Sustainability^d^
Rodríguez-Calderón et al.	2019	Article	Colombia	F	All	n/a
Balslev et al.	2010	Article	Peru	F	HF; MA	Unsustainable
Fadiman	2008	Article	Ecuador	F	MA	n/a
Coomes	2004	Article	Peru	F	MA	Unsustainable
O’Neill et al.	2001	Article	Peru	F	All	n/a
Gray et al.	2015	Article	Ecuador	F	All	Variable
García et al.	2013	Article	Colombia	F; M	MA	Sustainable
García et al.	2016	Article	Colombia	F	MA	Unsustainable
Kalliola and Flores	2011	Article	Peru	F	HF	Sustainable
de la Torre et al.	2011	Article	Colombia, Ecuador, Peru, Bolivia	n/a	All	n/a
Phillips et al.	1994	Article	Peru	F	All	n/a
Prance et al.	1987	Article	Bolivia	F	All	n/a
Rodríguez and Maldonado	2009	Article	Colombia	F	All	n/a
Gavin and Anderson	2007	Article	Peru	F	All	n/a
Willem et al.	2019	Article	Peru	F	HF	Variable
Quaedvlieg et al.	2014	Article	Peru	F	HF	n/a
Pyhälä, et al.	2006	Article	Peru	F	All	n/a
Bussmann and Sharon	2014	Article	Peru; Ecuador	F	ME	n/a
Bussmann et al.	2008	Article	Peru	S	ME	n/a
Duchelle	2007	Article	Ecuador	F	All	n/a
Thomas et al.	2017	Article	Peru	F	HF	n/a
Ramirez	2005	Article	Colombia	F	ME	n/a
Álvarez Salas et al.	2016	Article	Colombia	F	HF	n/a
Cronkleton et al.	2012	Article	Bolivia	F	HF; MA	Unsustainable
Coomes and Burt	2001	Article	Peru	F	FU	Variable
Duchelle et al.	2011	Article	Bolivia	F	HF	n/a
Guariguata et al.	2008	Article	Bolivia	F	HF; MA	n/a
Kvist et al.	2001	Article	Peru	F	All	Variable
Pacheco and Cronkleton	2008	Technical report	Bolivia	F	HF	n/a
Nebel	2001	Article	Peru	F	All	n/a
Duchelle et al.	2012	Article	Peru; Bolivia	F	HF; MA	Variable
Vennetier et al.	2012	Technical report	Bolivia	D	HF	n/a
De Jong et al.	2000	Article	Bolivia; Peru	n/a	All	n/a
Vallejo et al.	2016	Article	Colombia	F	HF	Unsustainable
Hoch et al.	2009	Article	Peru; Ecuador; Bolivia	F	MA; All	Variable
Vallejo et al.	2014	Article	Colombia	F	HF	Unsustainable
Gavin	2009	Article	Peru	F	All	n/a
Herrero-Jáuregui et al.	2013	Article	Colombia, Ecuador, Peru, Bolivia	F	HF; ME; MA; EU	n/a
Isaza et al.	2017	Article	Colombia	F	HF	Unsustainable
Rodríguez et al.	2018	Article	Colombia	S	All	Unsustainable
Svenning and Macía	2002	Article	Ecuador	F	MA	Variable
Fadiman	2019	Article	Ecuador	F	MA	Unsustainable
Weigend and Dostert	2005	Bulletin	Peru	X	ME	Sustainable
Kiehn	2004	Article	Ecuador	F	All	Variable
Argüello and Aguilar	2006	Bulletin	Ecuador	G	ME	Sustainable
Horn et al.	2012	Article	Peru	F	HF	Unsustainable
Bennett	2002	Article	Ecuador	F	All	n/a
Sælemyr	2004	Article	Ecuador	S	All	Sustainable
Manzi and Coomes	2009	Article	Peru	F	HF	Sustainable
Horn et al.	2018	Article	Peru	F	HF	n/a
Bruiton	1999	Technical report	Ecuador	n/a	ME	n/a
Hofstede et al.	2011	Book chapter	Ecuador	S; F	All	Sustainable
Cuoco and Cronan	2009	Article	Ecuador	F	SU	Unsustainable
Mesa-C and Galeano	2013	Article	Colombia	F	All	Unsustainable
Laureto and Cianciaruso	2017	Article	Colombia	n/a	All	n/a
Janni and Bastien	2000	Article	Bolivia	F	ME	n/a
Marshall et al.	2006	Technical report	Bolivia	F	All	Variable
Morsello et al.	2012	Article	Bolivia	F; G	MA	Variable
Newton et al.	2006	Article	Bolivia	F	All	Variable
Nuzzo and Aubertin	2007	Article	Bolivia	D	MA	n/a
Pedersen and Skov	2001	Article	Ecuador	n/a	All	Sustainable
Paneque-Gálvez et al.	2018	Article	Bolivia	F; G	All	n/a
Reyes-García et al.	2011	Article	Bolivia	F; G	All	Variable
Reyes-Garcia et al.	2007	Article	Bolivia	F; G	All	Variable
Sosnowska et al.	2015	Article	Peru	F	All	Sustainable
Thomas et al.	2011	Article	Bolivia	D	FU	Unsustainable
Camara-Leret et al.	2014	Article	Colombia, Ecuador, Peru, Bolivia	F; S	All	n/a
Stoian	2004	Book chapter	Bolivia	F	HF	Unsustainable

^a^Where studies were undertaken in multiple countries, only those in the Andean Community are listed

^b^Defined as per Olson et al. (2001). Abbreviations: F, Tropical and subtropical moist broadleaf forests; D, Tropical and subtropical dry broadleaf forests; G, Tropical and subtropical grasslands, savannas, and shrublands; S, Montane grasslands and shrubland; X, Deserts and xeric shrublands; M, Mangroves; n/a biome type was not relevant

^c^See Table 1. ‘All’ indicates that all plant uses were characterized rather than focusing on particular categories

^d^Sustainability of harvesting regime or conservation intervention, based on results and conclusions of the study. ‘Variable’ if sustainable harvesting occurred only under certain contexts; ‘n/a’ if no relevant assessment was undertaken

### Characteristics of studies

**Fig. 2:**
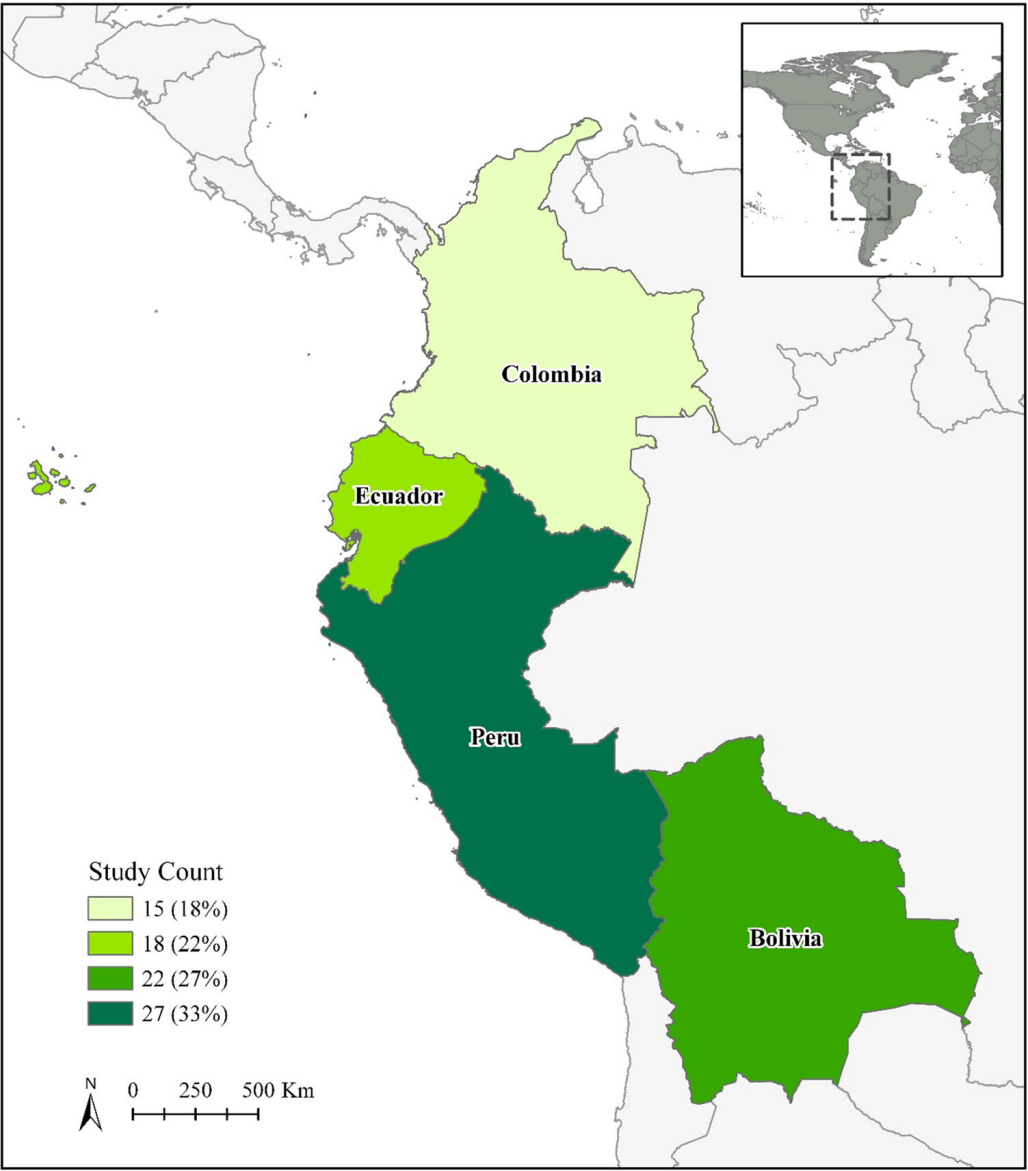
Distribution of studies on the conservation and management of useful wild plant species in the Andean Community (*n *= 68) (studies undertaken in ≥ 2 countries counted multiple times)

Publication year ranged from 1987 to 2019, with 96% of studies published since 2000 (Fig. S1). There was a relatively even split in the number of studies undertaken across the four Andean Community countries (*χ*^2^ = 3.9, df = 3, *p* = 0.267) (Fig. 2). Study number in Colombia has increased the most rapidly in recent years, with 57% of the 21 studies published since 2013 at least partially undertaken there.

The number of studies was unevenly distributed across biome types (*χ*^2^ = 189.9, df = 6, *p* < 0.001). Most were undertaken in tropical and subtropical moist broadleaf forests (74%), as defined by Olson et al. (2001) (Table 2; Fig. S2). These were primarily conducted in the Southwest Amazon moist forests ecoregion, which extend across the Andean countries of Peru and Bolivia.

**Fig. 3:**
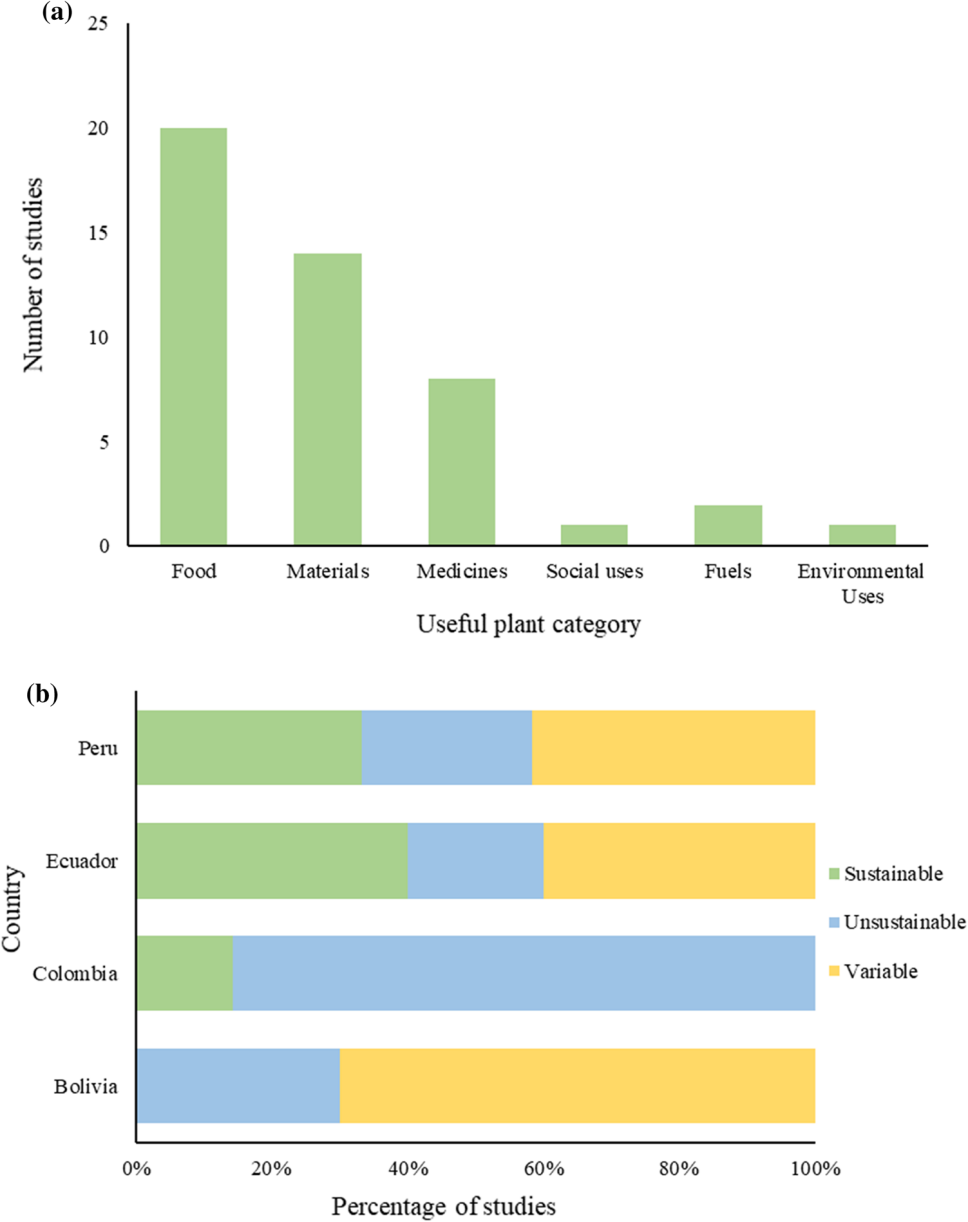
**a** Distribution of the number of studies across useful plant categories (excluding studies with no focus categories (*n *= 29) and counting studies with ≥ 2 focus categories multiple times). There was a significant difference from the expected mean count of 7.7 (*χ*^2^ = 40.9, df = 5, *p* < 0.001). **b** Percentage of studies in each country which were classified as showing sustainable, unsustainable, or variable outcomes. Studies with no relevant assessments are not shown (*n *= 33) and studies undertaken in ≥ 2 countries are counted multiple times. There was a significant difference in the proportion of studies with each outcome type between countries (*p* < 0.05, Fisher’s exact test)

Studies most commonly investigated all plant uses (*n *= 32, 47%), often in the context of non-timber forest products (NTFPs). Where specific uses were focused on, this was unevenly distributed (*χ*^2^ = 40.9, df = 5, *p* < 0.001); ‘human food’ (*n* = 20) and ‘materials’ (*n *= 14) were most commonly investigated (Table 2; Fig. 3a). Most studies did not focus on specific taxa, instead investigating all species within the study context (*n *= 37, 54%). Where this was not the case, palms (Areceae) were the most frequently studied family (19 records investigated individual palm species or the family) and the Brazil nut (*Bertholletia excelsa* Bonpl.) (*n *= 9) was the single most common case study species.

Thirty-six studies included an assessment of harvesting sustainability. Most indicated that sustainable long-term harvesting was not achieved (*n *= 14, 39%; Table 2) but there was a relatively even split between conclusions of whether harvesting was sustainable, unsustainable, or context-dependent (*χ*^2^ = 1.2, df = 2, *p* = 0.558). Nine instances of sustainable harvesting or successful management were documented, and 13 studies reported sustainable harvesting under certain circumstances (Weigend and Dostert 2005; Manzi and Coomes 2009). There were national differences (*p* < 0.05, Fisher’s exact test). Colombia had the highest percentage of studies reporting unsustainable outcomes (86%), while Bolivia, Peru, and Ecuador had more comparable results (*p* > 0.05 when Colombia removed from analysis), with 30, 25, and 20%, respectively (Fig. 3b).

#### Narrative synthesis

The studies reviewed included assessments of whether case studies of existing plant harvest were sustainable (Coomes 2004; Mesa-C and Galeano 2013); evaluations of conservation interventions (Horn et al. 2012; García et al. 2013), laws and policies (Guariguata et al. 2008; Duchelle et al. 2012); impacts of certification schemes (Pacheco and Cronkleton 2008; Quaedvlieg et al. 2014); attempts to establish new NTFPs (Cuoco and Cronan 2009; Vennetier et al. 2012); and more general reviews (O’Neill et al. 2001).

Despite the differences in study types, key themes emerged related to our research questions. Certain drivers of unsustainable harvesting and the loss of useful wild plant species arose repeatedly, and these factors were often also the focus for conservation and management recommendations and interventions. We categorized the drivers for useful plant loss and recommendations for sustainable harvesting as related to five key themes: plant biology; land tenure; knowledge, resource, and capacity; economics and market pressures; institutional structures, policy, and legislation.

#### Plant biology

The biological characteristics of species were frequently highlighted as important determinants of sustainable plant use across the studies reviewed. This includes plants’ regeneration capacity (De Jong et al. 2000), population size and density (Horn et al. 2012), and the habitat characteristics of harvesting locations (Svenning and Macía 2002).

Insufficient biological information on useful plant species has been cited as a driver of unsustainable harvesting, making it difficult to determine conservation status and appropriate management (Bennett 2002). Authors therefore highlighted the need to improve biological knowledge as a key conservation recommendation (Bruiton 1999; Svenning and Macía 2002; Stoian 2004; Marshall et al. 2006; Isaza et al. 2017). The basic biology, growth rates, and cultivation potential of *Krameria lappacea* (Dombey) Burdet & B.B.Simpson were investigated by Weigend and Dostert (2005), who successfully designed a local management plan for this medicinal and dye plant in Peru.

Certain plant traits are more likely to result in unsustainable use. Due to differences in survival probabilities and growth rates, models projected that palm fruit harvesting by Amazonian communities in Colombia have led to declining *Euterpe precatoria* Mart. populations, while *Mauritia flexuosa* L.f. remains stable (Isaza et al. 2017). However, favorable biological traits do not guarantee conservation success. The extraction of palm hearts, an NTFP with a large international market, drives the destructive felling of solitary palm species (single stemmed), with local extinctions of *Euterpe edulis* Mart. caused by overharvesting (Galetti and Fernández 1998). Non-destructive harvesting of caespitose palms (multiple stemmed) is possible and has been promoted as an opportunity for sustainable resource use (Stoian 2004), yet overharvesting remains a problem (Vallejo et al. 2014).

#### Land tenure and access rights

Individual and community decisions on natural resource management are significantly affected by land tenure—the way in which rights to use land and associated responsibilities are granted (FAO 2002).

Overharvesting of the Chiquitania almond *Dipteryx alata* Vogel was recorded on effectively open access ‘community lands’ following its commercialization in Bolivian communities (Vennetier et al. 2012). Similarly, *E. oleracea*’s location on communal land in Colombia contributed to unsustainable harvesting (Vallejo et al. 2016). There are ongoing debates around the sustainability implications of property regimes. Superficially, these two cases reflect Hardin’s much-cited ‘tragedy of the commons’ concept (1968), now widely recognized to have conflated open access and unsustainable extraction with sustainably managed common property regimes (Ostrom 1990). For instance, the depletion of *E. oleracea* was not solely due to its presence on communal property. A shift from harvesting palm hearts primarily for local consumption to harvesting for international markets changed the harvesting conditions generating economic dependence on the resource and leading to indiscriminate felling driven by income pressure (Vallejo et al. 2016).

Research assessing NTFP commercialization across Bolivia and Mexico concluded that no land tenure type (open access, community-run or private) necessarily prevents or creates overexploitation (Marshall et al. 2006). Many of the NTFPs studied were successfully harvested from community-run land when organizational structures were in place. This is echoed by Nebel (2001), who highlighted that sustainable use of common land requires strong institutional and organizational systems.

Land tenure issues can exist at the community as well as individual level. Policies across the Andean Community promote land clearance to gain formal property rights, often ignoring traditional plant uses and leading to colonization and loss of habitats used by indigenous communities with no formal land rights (Phillips et al. 1994; Kiehn 2004). Reporting on a sustainable management program for the moriche or aguaje palm *M. flexuosa* in Peru, Manzi and Coomes (2009) highlight that a key factor in its success was securing land tenure rights for the community. The necessity of clearly defined institutions and property rights for sustainable collective management is supported by Pyhälä et al. (2006).

The way in which land rights are determined also affects outcomes. “Bottom-up” approaches motivated by social movements and local governments lead to more successful long-term management and conservation than formalized, state-driven definitions of property (Duchelle et al. 2011). However, legal and administrative regulations can act as a barrier to bottom-up approaches, with rural stakeholders unable to access relevant information or with limited organizational experience (Marshall et al. 2006; Horn et al. 2012).

#### Knowledge, resource and capacity

The role of indigenous groups and other traditional peoples in managing a range of global habitats has been increasingly highlighted in conservation discourse (Bussmann 2002). This role is often underpinned by traditional ecological knowledge (TEK)—a culturally transmitted body of place-based belief, practice, and knowledge on relationships with the environment (Berkes 2017).

The use of wild plants is often linked to indigenous communities and TEK. Paneque-Gálvez et al. (2018) found a strong association between ethnobotanical knowledge and forest conservation in Tsimane’ Amerindian communities in Bolivia; villages with higher overall TEK may retain more ancestral beliefs linked to forest protection and may harvest more efficiently as more experienced foragers. Many authors therefore recommend the conservation, enhancement, and integration of TEK into management plans for wild plants (Ramirez 2005; Reyes-García et al. 2011; Cámara-Leret et al. 2014; Sosnowska et al. 2015).

However, destructive harvesting also exists in indigenous communities (Balslev et al. 2010; Fadiman 2019). Traditional methods may become unsustainable as extraction increases with market integration (Marshall et al. 2006) and resource availability decreases with land-use change. Additionally, alternative harvesting techniques can require more time, labor, and specialist tools than felling (Pedersen and Skov 2001; Manzi and Coomes 2009). Long-term plant population trends can also be difficult to determine. García et al. (2016) found that community management thought to increase production of *Astrocaryum* palm fibers actually led to long-term population declines.

Education of local users and the provision of training and tools for sustainable harvesting is often included in conservation recommendations (Thomas et al. 2011), with mixed results. Following an NGO-driven management program for moriche palm in Peru, Manzi and Coomes (2009) reported positive changes in community attitudes and practices. However, a similar program in different communities found that destructive harvesting continued (Horn et al. 2012), partly due to limited organizational experience at the community level.

Recommendations for education and training therefore go beyond practical plant management. Building capacity to develop a community’s organizational structures and understanding of market processes may be necessary (Manzi and Coomes 2009; Horn et al. 2012) alongside funding for tools and resources. Education of other stakeholders have also been recommended. This includes highlighting the importance of useful plant species among different forest users and educating international consumers on the practices involved in extraction (Bruiton 1999; Guariguata et al. 2008; Vallejo et al. 2016).

#### Economics and market pressures

The concept of conservation-through-use has led to economically driven conservation efforts, including attempts to create new markets for useful plant species or increase the value of already commercialized products (Arnold and Pérez 2001). However, commercialization is complex, with a range of factors affecting conservation outcomes.

Numerous barriers exist in accessing or developing markets for plant products. Poor infrastructure and distance to physical markets were a major factor in the failure to commercialize mocora palm products (*Astrocaryum standleyanum* L.L.Bailey) in Ecuador (Fadiman 2008). Creating viable new markets is difficult without investment in product promotion (Vennetier et al. 2012) and there is commonly a lack of market information among resource harvesters (Marshall et al. 2006). There are examples of newly commercialized NTFPs contributing to the reduction of rural poverty. However, in 75% of the 19 case studies assessed by Marshall et al. (2006), some overexploitation was observed.

The value of NTFPs is not always enough to prevent land-use change and timber extraction, even when commercialized (Marshall et al. 2006; Pyhälä et al. 2006; Quaedvlieg et al. 2014). Incomes are susceptible to market fluctuations (Bennett 2002; Stoian 2004; Nuzzo and Aubertin 2007). Additionally, the economic benefits derived from plant resources are often inequitably shared, disproportionately benefitting those higher up in the value chain (De Jong et al. 2000; Willem et al. 2019). This can undermine conservation outcomes, which are dependent on local resource harvesters maintaining sustainable livelihoods (Cuoco and Cronan 2009; Willem et al. 2019).

Certification schemes have been recommended to increase product value, sustainability, and harvester welfare (Rodriguez and Maldonado 2009; Kalliola and Flores 2011). Certification among Brazil nut harvesters in Bolivia enabled access to less volatile markets and formed associations, increasing political empowerment (Pacheco and Cronkleton 2008; Quaedvlieg et al. 2014). However, there are substantial barriers to gaining certification, making it difficult without NGO support. Additionally, poor schemes exist which can mask the realities of unsustainable harvesting (Vallejo et al. 2016).

Investment in local product-processing or business partnerships are another way to create value. However, Morsello et al. (2012) concluded that while these approaches can be successful, neither necessarily improved conservation or wellbeing among communities in Bolivia and Brazil. Outcomes are dependent on the context of the trade-offs involved and truly supportive public–private partnerships are difficult to establish (Nuzzo and Aubertin 2007). While many plant species contribute to the livelihoods and wellbeing of rural communities, claims of their economic potential can be difficult to realize (Nebel 2001).

#### Institutional structures, policy, and legislation

Legislation and policies relevant to conservation and wild plant use differ between the countries of the Andean Community. However, studies in all four nations report issues with current institutional structures. A review of regulations for the extraction and trade of NTFPs in Colombia, Ecuador, Peru, and Bolivia highlighted inconsistencies, contradictions, high administrative costs, and lack of implementation (de la Torre et al. 2011).

This has important conservation consequences. Inadequate regulation or poor implementation can lead to overharvesting or create conflict with other land-use activities (Willem et al. 2019). Overly complicated rules can make legal plant harvesting unviable for local producers, creating unregulated informal markets (Marshall et al. 2006; de la Torre et al. 2011). Meanwhile, contradictory and incoherent laws make it difficult to develop appropriate management (Bruiton 1999).

Numerous recommendations have been made at the international, national, and community levels to support sustainable plant use. Internationally, Laureto and Cianciaruso (2017) recommend incorporating economic and cultural importance in biodiversity assessments to ensure useful plants are included in conservation measures. More knowledge-sharing between countries on effective state policies and practices would also be beneficial (Bruiton 1999).

The importance of policies that recognize multiple land uses has been highlighted (Duchelle et al. 2012; Herrero-Jauregui et al. 2013; Willem et al. 2019). Currently, even high-value resources such as Brazil nuts are not integrated into policies for other uses such as timber extraction, creating conflicts, and trade-offs (Guariguata et al. 2008; Cronkleton et al. 2012). This requires negotiation and knowledge exchange between different sectors and government agencies. Further recommendations include supporting plant use as part of more diversified rural policies, incentivizing the study of sustainable harvesting, and specific policy creation to promote sustainable plant use and commercialization (Marshall et al. 2006). Removing market barriers indirectly has also been suggested, such as encouraging credit institutions (Newton et al. 2006).

Many studies highlight the need to include local resource harvesters in policy development. This would help to address legal discrimination against smallholders and communities (Duchelle et al. 2012). Additionally, state-driven regulation is often less sustainable than approaches which empower local stakeholders (O’Neill et al. 2001; Cronkleton et al. 2012). A study comparing Brazil nut concessions inside and outside a protected area found that the inclusion of concessionaires in decision-making within the reserve led to better, more sustainable performance than outside, where intervention focused on punitive measures (Willem et al. 2019). At the community level, technical and financial support could help gain relevant harvesting permits and understand regulations (Marshall et al. 2006).

While protected area establishment is a common state-driven conservation intervention, we found few examples of reserves designated for useful plants. An exception is the Condor Bioreserve—four protected areas in Ecuador which jointly aim to conserve and protect natural resources, promote sustainable economic initiatives, and develop funding mechanisms with the private sector (Kiehn 2004). Management conflicts with plant users have been reported in some protected areas, such as the Podocarpus National Park (PNP) in Ecuador (Sælemyr 2004). Conversely, the establishment of Allpahuayo-Mishana National Reserve (RNAM) in Peru allowed the continuation of local resource use, only preventing non-residents from harvesting. This saw mixed results, with the authors concluding that protected area managers should work with local communities to understand livelihood strategies and jointly identify management rules (Pyhälä et al. 2006).

## Discussion

We identified 68 studies that evaluated the sustainability of wild plant use in the Andean Community and characterized their key traits. The majority were published since 2000 (Table 2). This reflects the increasing focus on conservation in ethnobotanical research (Balick 1996) and the increasing recognition of traditional natural resource use in conservation discourse (Cooney 2007).

The focus on tropical moist forests can be partly attributed to the fact that this biome covers a large proportion of the countries studied (Olson et al. 2001), is highly productive, and is rich in biological and cultural diversity. Palms play a key ecological role in tropical and subtropical ecosystems and are recognized as a fundamental resource in traditional communities (Laureto and Cianciaruso 2017). This is reflected in the number of studies which focused on useful palm species, with this focus potentially also contributing to the high number of studies in tropical forest biomes.

The Brazil nut was the single most common case study species, explained by its status as one of the most economically important NTFPs of the Amazonian region (Kalliola and Flores 2011). The international markets associated with this resource, its contribution to livelihoods, and national policies to support sustainable harvest, provides an example of a useful plant species motivating conservation action and supporting development. However, even for Brazil nut harvesting, the sustainability of outcomes is extremely variable (Willem et al. 2019).

The number of studies which reported unsustainable or sustainable use was relatively even, with many also concluding that sustainable harvests are only possible under certain conditions (Table 2). However, there were national differences in these findings (Fig. 3b). The conditions for achieving successful conservation-through-use are evidently complex (Coomes 2004; Vallejo et al. 2016).

### The importance of context

Drivers of sustainable and unsustainable plant use and targets for recommended conservation and management actions were highlighted across the literature reviewed. We identified five key, reoccurring themes within which most of these factors could be grouped: plant biology; land tenure; knowledge, resource and capacity; economics and market pressures; and institutional structures, policy, and legislation.

These themes arose across studies of different plant species, use categories, communities, and countries in the Andean Community, highlighting their importance in determining the sustainability of wild plant use. Despite this apparent consistency, there was high variability in management outcomes, with even the most repeated conservation recommendations being unsuccessful in certain scenarios. Many authors highlight the need for management plans to be flexible and context-specific (Svenning and Macía 2002), to consider the combination of biophysical, social, and institutional conditions.

The importance of context cut across all five identified themes. For instance, land tenure solutions displayed context-dependent outcomes and were dependent on historical context (Duchelle et al. 2011).

The need for policy and legislative changes was frequently highlighted. This is a global issue, with national laws and policies on NTFPs usually lacking coherence (Laird et al. 2010). However, as Newton et al. (2006) concluded, the impact of policy changes is dependent on factors such as local capacities and the resource itself. Knowledge developed through long-term global experiences on sustainable natural resource use should be adapted to regional and local contexts to help develop policies, regulations, and conservation and management programs. However, such policy development is only likely to occur if national governments and society first acknowledge the importance of sustainable use. While global principles and agreements increasingly highlight the importance of sustainability (Convention on Biological Diversity 1992; CBD 2020b), they can only be effective if adapted to national, regional, and local contexts (Boedhihartono et al. 2018). Wild-harvested plant products have rarely been recognized or included in policy and legislation in the Andean Community, despite their importance for livelihoods across the region. Understanding national priorities, such as development, is therefore an important first step in adapting the framing of international principles and agreements to fit with national agendas and therefore increase the likelihood that sustainable plant use is recognized by governments as socially, ecologically, and economically important. In this sense, acknowledging the factors involved at different geographic scales is crucial, not just to develop relevant, bottom-up methods for sustainable use on the ground, but also to promote the recognition of the importance of the issue in political agendas.

Beyond the factors grouped within the five key themes identified, several other recommendations were made for the conservation of useful wild plant species in certain contexts. This included the cultivation of currently wild-harvested plants to prevent the degradation of wild populations (Thomas et al. 2011; García et al. 2016) and planting to regenerate degraded habitats (García et al. 2016).

### National political and economic differences

Despite some comparable characteristics in institutional structures and policies cross the Andean Community, 86% of studies in Colombia indicated unsustainable harvesting, compared to 20–30% in Ecuador, Peru, and Bolivia (Fig. 3b). Differences in political approaches to biodiversity conservation and development and economic structure may partly explain these differences.

International treaties such as the CBD’s Nagoya Protocol and the International Treaty on Plant Genetic Resources for Food and Agriculture (FAO 2020) are relevant to plant use. All four Andean Community countries are signatories to both; however, Colombia is the only country that has not ratified either (CBD 2020a). These differences may be linked to the varying levels of national importance the bioeconomy plays. Defined globally as “the production, utilization and conservation of biological resources (…) in all economic sectors” (Rodríguez et al. 2019), the value of bioeconomy-related exports between 2010 and 2015 accounted for 38.3% of Ecuador’s total exports, compared to 14.7% of Colombia’s (Rodríguez et al. 2017). Conversely, Colombia and Bolivia, where the lowest proportion of studies reported sustainable plant use, were the countries where fossil fuels and minerals accounted for the greatest proportion of export value. Conservation-through-use arguments may therefore be relevant at a national level, with bioeconomic benefits incentivizing policies promoting sustainable practices. This has led to programs such as the United Nations’ (UN) BioTrade Initiative, promoting trade and investment in biodiversity to advance sustainable development. Encouragingly, Bolivia, Ecuador, Colombia, and Peru are all members implementing national programs (UN Conference on Trade and Development, Undated).

### The importance of collaboration and conservation

The need for increased collaboration and dialogue between stakeholders was evident across all the themes. Ethnobotany and conservation are inherently interdisciplinary, requiring specialists from numerous fields to provide a broad base for conservation and management. Meanwhile, local NGOs can be crucial in providing a link between scientists and local participation (Bussmann 2002).

The importance of actively working with communities to enable bottom-up approaches was repeatedly highlighted (Marshall et al. 2006; Duchelle 2007). This is supported by the degradation and marginalization thesis, with overexploitation shown to increase under conditions where local communities are marginalized or subject to disruptive social change from external intervention (Robbins 2012). A large proportion of tropical forests are community-owned and it is vital to recognize that effective management and sustainable use is ultimately dependent on local resource users having the flexibility to manage habitats themselves (Alexiades et al. 2013).

The various demands on natural habitats necessitate multi-stakeholder dialogue and interventions which recognize all users (Guariguata et al. 2008). This includes balancing use with conservation interventions such as the establishment of protected areas. However, very few of the studies we reviewed reported on protected areas that specifically target the conservation of useful plant species. This finding is reflected globally. In a study assessing the comprehensiveness of the conservation of useful wild plants, Khoury et al. (2019) reported that fewer than 3% of species are sufficiently conserved, with gaps in ex situ conservation found to be even greater than in situ efforts.

Although conservation gaps continue to exist, recent progress has been made in conservation-related research and practice to improve international in situ conservation of useful plant species. The Important Plant Area (IPA) program—established to identify and protect a network of best sites for plant conservation in the world—was updated in 2017 to include socially, economically, or culturally valuable species in its criteria for site identification. While this is yet to be widely implemented, recent national examples of protected areas for useful plant species exist. The *Santuario de Flora Plantas Medicinales Orito Ingi*-*Ande* was declared as part of Colombia’s system of National Natural Parks in 2008 (IUCN 2008)—the only protected area for medicinal plants in the country. The Sanctuary is located within territories used by the Kofanes indigenous community, who first proposed the concept and were crucial in the characterization of species within the reserve. Collaboration was therefore crucial in this process, involving local community leaders, various Colombian ministries, academic institutions, and NGOs (IUCN 2008).

## Conclusions and recommendations

Our review demonstrates that the sustainability of wild plant use is a complex topic which has attracted research and conservation efforts across the Andean Community. With unprecedented levels of global biodiversity loss and the high cultural and biological diversity of the region, the importance of sustainable use is of international importance. Overharvesting can negatively impact social and economic wellbeing, drive wildlife loss, and cause the loss of key ecosystem functions and services. Based on the key themes which emerged from this review, we recommend the following actions for researchers, conservationists, and local governments involved in the conservation and management of useful wild plant species:

(i) Engage and collaborate. Ethnobotany and conservation are interdisciplinary topics, with a need for stakeholders from different sectors and research disciplines to engage in open dialogue and work together to develop solutions to sustainably balance natural resource use and conservation (Bussmann 2002).
(ii) Involve local resource users. The ecological knowledge held by local and indigenous users must be recognized and fully incorporated into management and conservation plans (Ramirez 2005; Reyes-García et al. 2011; Cámara-Leret et al. 2014; Sosnowska et al. 2015). Only in this way can long-held traditional plant uses be balanced with the modern demands on natural resources to form long-term sustainable solutions which work within the unique local context.
(iii) Improve recognition of the importance of wild plant use. Despite being vital for millions of livelihoods, wild-harvested plant products have rarely been recognized or included in policy and legislation in the Andean Community and internationally. Raising awareness of their importance among policy makers and across society would be a vital first step in developing effective policies that recognize multiple land uses and support sustainable wild plant use.
(iv) Support studies on sustainable harvesting. Plant biology and habitat characteristics are important factors in sustainable harvesting rates and practices. Supporting and furthering knowledge of useful plant species is therefore vital in designing effective, context-specific management plans.

Though based on a review of literature from the Andean Community, these recommendations are of global relevance. Similar key themes have emerged across the world as conservation-through-use and bioeconomy-based approaches are increasingly applied to meet international conservation efforts which recognize the crucial human dimensions of conservation.

## Supplementary Information

Below is the link to the electronic supplementary material.

Electronic supplementary material 1 (PDF 223 kb)
