# Supplementary material for: Sustainability of wild plant use in the Andean Community of South America
Source: Ambio. 2021 Apr 16;50(9):1681–97. doi: 10.1007/s13280-021-01529-7 (PMC8285437; doi:10.1007/s13280-021-01529-7)
Supplement: Supplementary file 1 — Electronic supplementary material 1 (PDF 223 kb) [file 13280_2021_1529_MOESM1_ESM.pdf]

Ambio

Electronic Supplementary Material
This supplementary material has not been peer reviewed.

Title: **Sustainability of wild plant use in the Andean Community of South America**

Table S1. Search terms used in ISI Web of Science and Scopus databases. Search strings in English or Spanish were formed across the three categories using the Boolean operator AND. E.g. Colombia AND (“useful plant*” OR “underutilized plant*” OR “plant use”) AND conserve*

**Location**	**Useful plants**	**Conservation**
Colombia	useful plant* OR underutilized plant* OR plant use	conserv*
Ecuador	plant use	protect*
Peru	ethnobot*	sustain*
Bolivia	plant resource*	management
	medicin* plant	parque* nacional*
	food plant* OR edible plant*	sostenib*
	non-timber forest product*	gestión
	planta* útile* OR planta* subutilizada* OR uso de las plantas	
	etnobotán*	
	recurso vegetal	
	planta* medicin* OR planta* alimenticia*	
	planta* comestible	
	productos forestales no maderables	

Table S2. Categories included in the literature review data extraction table and their descriptions

Field	Description
Year	Year of publication.
Journal	Title of the journal, book, or bulletin in which the study was published
Reference type	Type of publication, included: (a) Article – published in peer-reviewed journal, (b) Book Chapter, (c) Technical report – issued by governmental or non-governmental organisations, (d) Bulletin – issued in newsletters by special interest groups or societies.
Language	Language in which full text was published.
Study counties	The countries in which the study took place or focused on.
Biome	The biomes in which the study took place or focused on. We categorized this by comparing the study location against mapped data from Terrestrial ecoregions of the world: a new map of life on Earth (Olson et al, 2001). Categorized as n/a where biome was not relevant e.g. review of legislation across the study countries.
Ecoregion	The ecoregions in which the study took place or focused on. We categorized this by comparing the study location against mapped data from Terrestrial ecoregions of the world: a new map of life on Earth (Olson et al, 2001).
Study methods	The types of methods described in the study's methods section.
Useful plant categories	The plant uses focused on in the study. We categorized this according to the categories described in the World Checklist of Useful Plant Species (Diazgranados et al., 2020), based on the Level 1 States defined by Cook, 1995. Where studies did not focus on specific use categories, this was classified as 'all'.
Focal taxa	The plant species or family of focus in the study, where applicable. Classified as n/a where the study did not focus on specific taxa.
Social communities	The groups or communities which were the focus of the study.
Conservation relevance	A description of the study's relevance to conservation or sustainable management based on the authors' description of aims and objectives.
Conservation outcomes or recommendations	We summarised the key findings and recommendations from the study, focusing on relevance to the review's main research questions.
Key drivers	We categorized the main drivers of unsustainable management in the study as related to: plant biology (BI); land tenure (LT); knowledge, resource and capacity (KE); external threats (EXT); economic factors (ECO); institutional structures, legislation and policy (LP)
Key recommendations	We categorized the main recommendations for sustainable use identified in the study as related to: collaboration (COL); biological information (BI); traditional ecological knowledge (TEK); context-specific management (SPE); land tenure (LT); economic and market development (ECO); education and capacity building (KE); cultivation (CUL); policy, legislation and institutional structures (LP)
Sustainable harvesting / successful intervention	Based on the study results, we classified whether or not the outcomes of the useful plant harvesting or conservation intervention studied were sustainable as: (a) yes – existing sustainable harvesting or successful management / conservation intervention; (b) no – unsustainable harvesting or unsuccessful intervention; (c) variable – sustainable harvesting was found to occur only under certain contexts, or (d) n/a – no relevant results for this field.

Categorization of studies on the sustainability of wild plant use

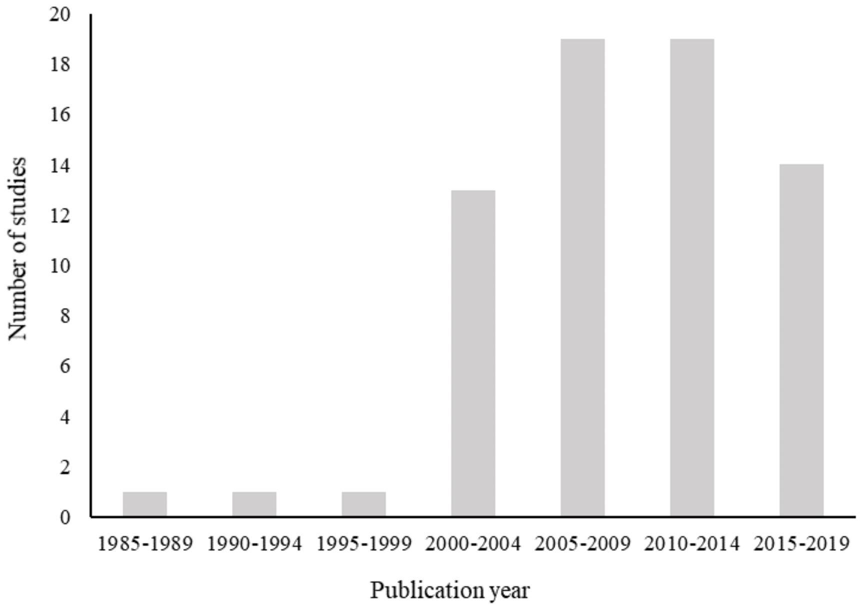

Figure S1. Distribution of the publication years of the 68 studies included in the review, ranging from 1987 to 2019. The number of studies published per half-decade during this period significantly differed (χ^2=44.1, df=6, p<0.001).

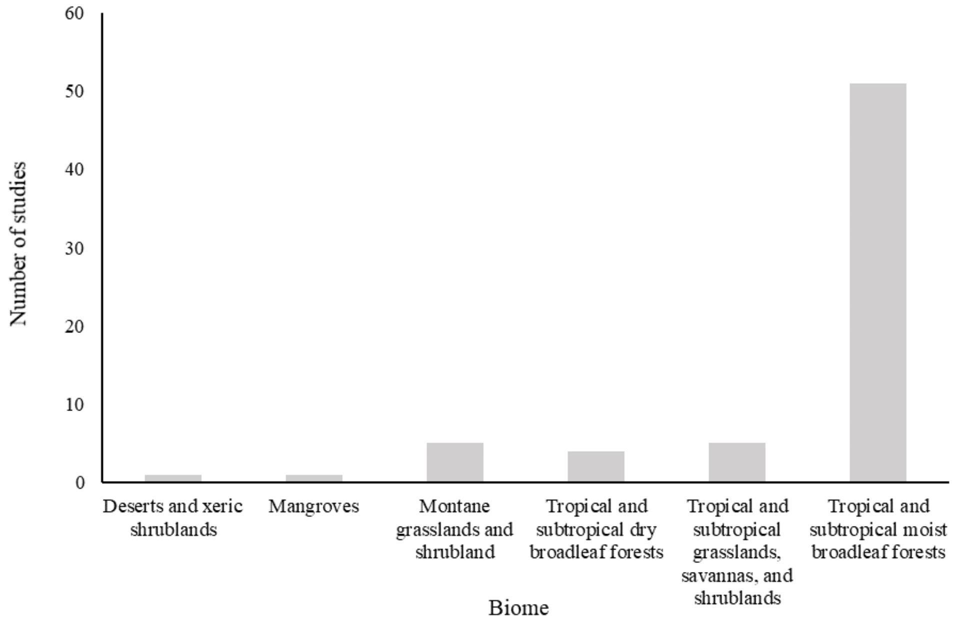

Figure S2. Distribution of the number of studies across biomes (excluding studies with no focus study location (n=5) and counting studies undertaken across ≥2 biomes multiple times). There was a significant difference from the expected mean count of 10.3 (χ^2=189.9, df=5, p<0.001).
